# Morphological and molecular study on *Yininemertespratensis* (Nemertea, Pilidiophora, Heteronemertea) from the Han River Estuary, South Korea, and its phylogenetic position within the family Lineidae

**DOI:** 10.3897/zookeys.852.32602

**Published:** 2019-06-05

**Authors:** Taeseo Park, Sang-Hwa Lee, Shi-Chun Sun, Hiroshi Kajihara

**Affiliations:** 1 National Institute of Biological Resources, Incheon, Korea National Institute of Biological Resources Incheon South Korea; 2 National Marine Biodiversity Institute of Korea, Secheon, Korea National Marine Biodiversity Institute of Korea Secheon South Korea; 3 Institute of Evolution and Marine Biodiversity, Ocean University of China, Yushan Road 5, Qingdao 266003, China University of China Qingdao China; 4 Faculty of Science, Hokkaido University, Sapporo 060-0810, Japan Hokkaido University Sapporo Japan

**Keywords:** *
Anguilla japonica
*, brackish-water invertebrates, freshwater invertebrates, Yellow Sea

## Abstract

Outbreaks of ribbon worms observed in 2013, 2015, and 2017–2019 in the Han River Estuary, South Korea, have caused damage to local glass-eel fisheries. The Han River ribbon worms have been identified as *Yininemertespratensis* (Sun & Lu, 1998) based on not only morphological characteristics compared with the holotype and paratype specimens, but also DNA sequence comparison with topotypes freshly collected near the Yangtze River mouth, China. Using sequences of six gene markers (18S rRNA, 28S rRNA, histone H3, histone H4, 16S rRNA, and COI), the phylogenetic position of *Y.pratensis* was inferred among other heteronemerteans based on their sequences obtained from public databases. This analysis firmly placed *Y.pratensis* as a close relative to *Apatronemertesalbimaculosa* Wilfert & Gibson, 1974, which has been reported from aquarium tanks containing tropical freshwater plants in various parts of the world as well as a wild environment in Panama.

## Introduction

An explosive proliferation of unidentified, brackish-water heteronemerteans was observed in the Han River Estuary, South Korea, in the spring of 2013. Our morphological observation of the Han River ribbon worms indicated that they represent *Yininemertespratensis* (Sun & Lu, 1998), a brackish-water heteronemertean known only by its original description from the Yangtze (Changjiang) River Estuary, China (for the nomenclature of the genus, see Sun and Lu 2008; Özdikmen 2009; Kajihara 2014). Outbreaks of *Y.pratensis* in the Han River Estuary were also observed in 2015, 2017, 2018, and 2019. Reportedly, the worms have caused severe damage (Lee 2015; Noh 2019) to local fisheries of glass eels, which are juveniles of *Anguillajaponica* Temminck & Schlegel, 1847, a valuable fishery resource in East Asian countries showing dramatic declines in recent years (Tzeng 1997; Tatsukawa 2003; Tseng et al. 2003). As causes for the eel declines, overfishing and habitat loss due to human activities (e.g., Chen et al. 2014) and oceanic–atmospheric factors such as changes in ocean circulation (Chang et al. 2018) have been suggested. To what extent the nemerteans have been contributing to the anguillid declines is not known. For glass-eel fisheries, fishermen set long, conical nets on the estuarine bottoms with apertures directing downstream. At the end of each net, ascending catches are to be concentrated mostly during flood tide. In the 2015 bloom, more than 90% of catches were worms, with none to only a few eels that were dead (Lee 2015) probably due to yet-unidentified neurotoxic substances (Kwon et al. 2017) in worm mucus within the concentrated net catches. These neurotoxins might have been discharged from epidermal cells and contained in the secreted mucus (cf. Tanu et al. 2004; Asakawa et al. 2013). To our knowledge, this is the first record of damage to fisheries directly caused by nemertean outbreaks, although a potentially indirect case is known. At certain Alaskan localities in the 1983–1984 and 1984–1985 brooding seasons of the red king crab *Paralithodescamtschaticus* (Tilesius, 1815), a widespread outbreak of the decapod-egg-predatory nemertean *Carcinonemertesregicides*Shields et al., 1989, and possibly *Ovicidesparalithodis* Kajihara & Kuris, 2013 as well, caused high egg mortality (Kuris et al. 1991), which could have led to a subsequent decline in the red king crab population (e.g., Loher and Armstrong 2005). In addition, the milky ribbon worm *Cerebratuluslacteus* (Leidy, 1851) has been identified as an important threat to populations of the soft-shell clam *Myaarenaria* Linnaeus, 1758, which is one of the commercial bivalves in Atlantic Canada, although no outbreak has ever been reported for *C.lacteus* (cf. Bourque et al. 2001, 2002).

Facing a plethora of undescribed species with dwindling number of experts, some nemertean taxonomists agreed that taxonomic descriptions of ribbon worms will have to shift from traditional, internal-anatomy-based style to histology-free one with a combination of high-quality external images and molecular phylogeny (Strand and Sundberg 2011; Strand et al. 2014; Kajihara 2015; Sundberg et al. 2016). On the other hand, in the case of Heteronemertea, only about 10% of ~100 genera (Gibson 1995; Kajihara et al. 2008) have been represented by type species in terms of sequences for multi-locus analysis (Thollesson and Norenburg 2003; Andrade et al. 2012; Kvist et al. 2014, 2015). Logically, until the rest of ~90 genera are also represented in the same manner, examination of internal morphology will remain indispensable to genus-level identification (e.g., Chernyshev et al. 2018). Moreover, most heteronemertean genera currently diagnosed are non-monophyletic. This has been repeatedly pointed out in previous studies (e.g., Sundberg and Saur 1998; Schwartz 2009; Puerta et al. 2010; Hiebert and Maslakova 2015). Therefore, as many type species of genus-group names (such as *Yininemertes*) as possible should be placed in molecular phylogenetic context for proper application of genus names in many other species of heteronemerteans as long as Linnaean binominal nomenclature is employed.

In this paper, we report the identity of Han River nemerteans based on morphological characteristics in comparison to the type material of *Y.pratensis* as well as DNA barcoding data from the type locality. Also, we infer the phylogenetic position of *Y.pratensis* among Heteronemertea based on a multi-locus molecular analysis.

## Materials and methods

### Specimen collection and processing

**Figure 1.**
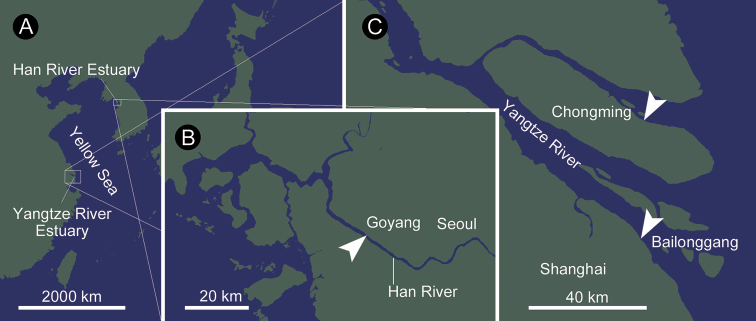
Maps showing sampling localities indicated by arrow heads. **A** The Han River and Yangtze River Estuaries are about 840 km apart from each other across the Yellow Sea **B** sampling locality in Goyang, Korea **C** two sampling localities, Chongming Island and Bailonggang, China.

**Figure 2.**
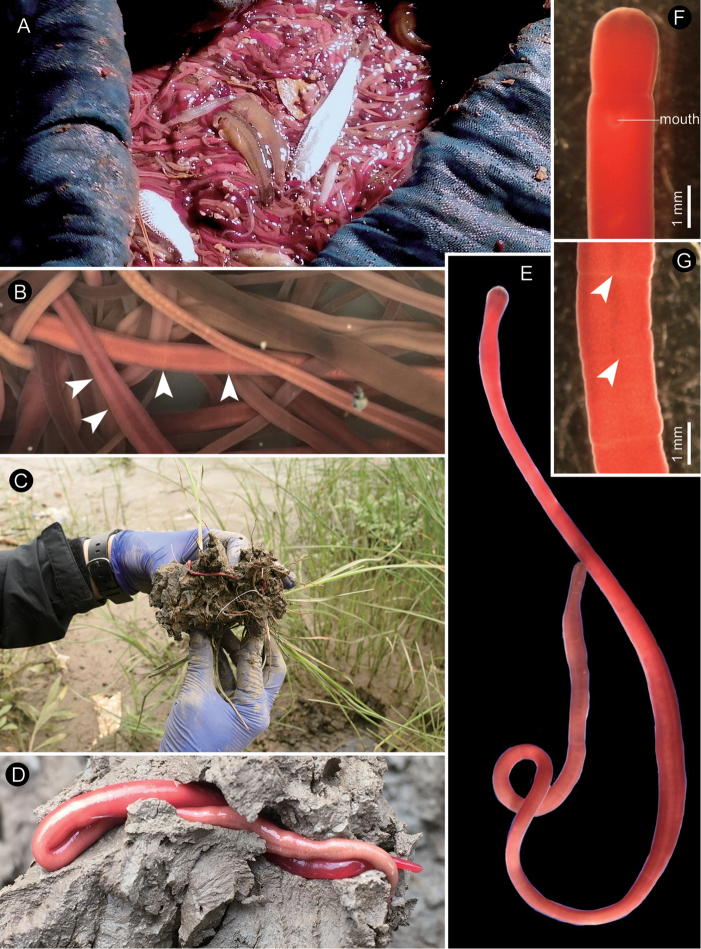
Photographs of *Yininemertespratensis* (Sun and Lu, 1998) taken in life. **A** A haul of a glass-eel net at the Han River Estuary, South Korea, on 6 April 2015 **B** magnification of a swarm of the same worms as in **A** from the Han River Estuary taken in the laboratory; arrow heads indicating the characteristic transverse narrow rings in the intestinal region **C** a specimen dug from clayey mud sediment with vegetation at Bailonggang in the Yangtze River Estuary, China, May 13, 2016 **D** a specimen dug from non-vegetated clay sediment at Chongming Island in the Yangtze River Estuary, China, 14 May 2016 **E** topotype from the Yangtze River Estuary showing an overview of whole specimen **F** topotype from China showing magnification of head, ventral view **G** topotype from China, magnification of intestinal region, showing the characteristic narrow transverse rings, indicated by arrow heads.

Approximately 700 individuals of ribbon worms were collected from local fishermen’s glass-eel nets for *Anguillajaponica*, set at about 37°36'08"N, 126°48'23"E, in Goyang, South Korea, approximately 40 km upstream of the mouth of the Han River (Figs 1A, B, 2A) on April 6, 2015 by TP. A total of 12 topotype specimens of *Y.pratensis* were collected at two sites in Shanghai by TP, Kwang-Soo Kim, Seul Yi, SS, and Guang Xi: i) Bailonggang, 31°15'40.0"N, 121°44'11.8"E, on May 13, 2016; and ii) Chongming Island, 31°34'39.4"N, 121°54'34.9"E, on May 14, 2016 (Figs 1A, C, 2C). Specimens from the Han River were anesthetized with 7% MgCl_2_ solution before fixed in either 7% neutral-buffered formalin for morphological observation (~300 individuals) or 100% ethanol for DNA extraction (~300 individuals). Of these 12 specimens collected from Shanghai, nine were fixed in 70% EtOH for DNA extraction while three were used for taking photographs. Anterior portion of one formalin-fixed specimen from the Han River was dehydrated in ethanol series, cleared in xylene, embedded in paraffin (melting point: 56–57 °C), and transversely sectioned at thickness of 8 µm. Serial sections were stained with Mallory’s trichrome method (Gibson 1994). Specimens were deposited in National Institute of Biological Resources Invertebrate Collection, Incheon, Korea (NIBR IV) and Invertebrate Collection of the Hokkaido University Museum, Sapporo, Japan (ICHUM) (Table 1). For comparison, the holotype (DH005A) and a paratype (DH005C) of *Y.pratensis* deposited in Ocean University of China, Qingdao, People’s Republic of China, were also examined.

**Table 1.** List of specimens identified as *Yininemertespratensis* (Sun & Lu, 1998) in this study with catalogue numbers at the National Institute of Biological Resources Invertebrate Section, Incheon, Korea (NIBR IV) and the Invertebrate Collection of the Hokkaido University Museum, Sapporo, Japan (ICHUM) as well as their sampling date and locality.

Catalogue number	Sampling date and locality	Remarks
NIBR IV 0000409587–0000409590	6 April 2015, Goyang, South Korea	> 300 individuals fixed in 10% formalin
NIBR IV 0000409591–0000409595	6 April 2015, Goyang, South Korea	> 300 individuals fixed in 100% EtOH
NIBR IV 0000409596–0000409617	6 April 2015, Goyang, South Korea	22 voucher specimens used for DNA extraction
NIBR IV 0000758851	13 May 2016, Bailonggang, China	1 specimen fixed in 70% EtOH
NIBR IV 0000758852	13 May 2016, Bailonggang, China	1 specimen fixed in 70% EtOH
NIBR IV 0000758853	13 May 2016, Bailonggang, China	1 specimen fixed in 70% EtOH
NIBR IV 0000758854	13 May 2016, Bailonggang, China	1 specimen fixed in 70% EtOH
NIBR IV 0000758855	13 May 2016, Bailonggang, China	1 specimen fixed in 70% EtOH
NIBR IV 0000758856	13 May 2016, Bailonggang, China	1 specimen fixed in 70% EtOH
NIBR IV 0000758857	13 May 2016, Bailonggang, China	1 specimen fixed in 70% EtOH
NIBR IV 0000758848	14 May 2016, Chongming, China	1 specimen fixed in 70% EtOH
NIBR IV 0000754958	14 May 2016, Chongming, China	1 specimen fixed in 70% EtOH
ICHUM 5259	6 April 2015, Goyang, South Korea	8 specimens fixed in 10% formalin
ICHUM 5260	6 April 2015, Goyang, South Korea	Serial transverse sections of the anterior portion of a specimen, Mallory trichrome, 36 slides.

### Molecular phylogeny

Small pieces of tissue taken from 22 specimens collected from the Han River and seven specimens from Yangtze River were used for total genomic DNA extraction using DNeasy Blood and Tissue Kit (Qiagen, Hilden, Germany) following the manufacturer’s instructions. Partial sequences of six gene markers (nuclear 18S rRNA, 28S rRNA, histone H3, and histone H4; mitochondrial 16S rRNA, and COI) were used for molecular analyses using the same primers published by Andrade et al. (2012). For PCR amplification, the following mixture was prepared in a total volume of 50 μL: 50 ng of template genomic DNA, 2.5 mM dNTPs, 5 μL of 10× Ex Taq™ buffer, 2 μL of each 10 pM primer, and 1 U (0.5 μL) of TaKaRa Ex Taq™ polymerase. Thermal cycling condition comprised an initial denaturation at 94 °C for 30 sec followed by 35 cycles of denaturation at 98 °C for 10 sec, annealing at 43–50 °C depending on primers for 30 sec, and extension at 72 °C for 1 min. A final extension step at 72 °C for 10 min was then followed. Amplified PCR products were sequenced using an ABI 3730 sequencer (Applied Biosystems, Foster City, CA, USA) from both directions. All sequences generated de novo in this study were deposited at GenBank (Table 2).

**Table 2.** GenBank accession numbers of sequences determined in the present study from voucher specimens of *Yininemertespratensis* (Sun & Lu, 1998) deposited in the National Institute of Biological Resources Invertebrate Collection, Incheon, Korea (NIBR IV).

NIBR IV	18S rRNA	28S rRNA	Histone H3	Histone H4	16S rRNA	COI
0000409596	KY274047	KY274069	KY274091	KY274113	KY274025	KY274003
0000409597	KY274048	KY274070	KY274092	KY274114	KY274026	KY274004
0000409598	KY274049	KY274071	KY274093	KY274115	KY274027	KY274005
0000409599	KY274050	KY274072	KY274094	KY274116	KY274028	KY274006
0000409600	KY274051	KY274073	KY274095	KY274117	KY274029	KY274007
0000409601	KY274052	KY274074	KY274096	KY274118	KY274030	KY274008
0000409602	KY274053	KY274075	KY274097	KY274119	KY274031	KY274009
0000409603	KY274054	KY274076	KY274098	KY274120	KY274032	KY274010
0000409604	KY274055	KY274077	KY274099	KY274121	KY274033	KY274011
0000409605	KY274056	KY274078	KY274100	KY274122	KY274034	KY274012
0000409606	KY274057	KY274079	KY274101	KY274123	KY274035	KY274013
0000409607	KY274058	KY274080	KY274102	KY274124	KY274036	KY274014
0000409608	KY274059	KY274081	KY274103	KY274125	KY274037	KY274015
0000409609	KY274060	KY274082	KY274104	KY274126	KY274038	KY274016
0000409610	KY274061	KY274083	KY274105	KY274127	KY274039	KY274017
0000409611	KY274062	KY274084	KY274106	KY274128	KY274040	KY274018
0000409612	KY274063	KY274085	KY274107	KY274129	KY274041	KY274019
0000409613	KY274064	KY274086	KY274108	KY274130	KY274042	KY274020
0000409614	KY274065	KY274087	KY274109	KY274131	KY274043	KY274021
0000409615	KY274066	KY274088	KY274110	KY274132	KY274044	KY274022
0000409616	KY274067	KY274089	KY274111	KY274133	KY274045	KY274023
0000409617	KY274068	KY274090	KY274112	KY274134	KY274046	KY274024
0000754958	KY274138	KY274140	KY274144	KY274146	KY274136	KY274142
0000758857	KY274137	KY274139	KY274143	KY274145	KY274135	KY274141

To assess phylogenetic affinity of the Han River nemerteans, maximum likelihood (ML) analysis and Bayesian Inference (BI) were carried out with 31 lineid heteronemertean species for which the aforementioned six gene sequences were available in public databases (Table 3). Outgroups were chosen to include *Baseodiscusmexicanus* (Bürger, 1893) and *B.unicolor* Stiasny-Wijnhoff, 1925 (cf. Andrade et al. 2012; Kvist et al. 2014). Sequence alignment was performed using MAFFT ver. 7 (Katoh and Standley 2013) with *E-INS-i* option for 18S, 28S, and 16S. For the protein-coding H3, H4, and COI, sequences were aligned straightforward without gaps. Sequences were edited and concatenated using MEGA ver. 5.2 (Tamura et al. 2011). Gaps and incompletely determined nucleotides accounted for 24.9% of the entire dataset of these sequences.

**Table 3.** GenBank accession numbers of sequences used in the present phylogenetic analysis (Histone H4 sequences indicated by asterisks (*) were kindly provided by Dr Sebastian Kvist).

	18S rRNA	28S rRNA	Histone H3	Histone H4	16S rRNA	COI	Reference
*Apatronemertesalbimaculosa* Wilfert & Gibson, 1974^a^	JF293030	HQ856860	JF277733	JF277666	JF277587	HQ848584	Andrade et al. (2012)
*Cerebratuluslacteus* (Leidy, 1851)	JF293044	HQ856857	JF277728	JF277653	JF277575	HQ848576	Andrade et al. (2012)
*Cerebratulusmarginatus* Renier, 1804	JF293042	HQ856858	JF277729	JF277652	JF277576	HQ848575	Andrade et al. (2012)
*Gorgonorhynchusalbocinctus* Kajihara, 2015	LC010650	LC010651	–	–	–	LC010649	Kajihara (2015)
Gorgonorhynchuscf.bermudensis Wheeler, 1940^b^	KF935300	KF935356	KF935412	*	KF935467	KF935517	Kvist et al. (2014)
*Kulikoviaalborostrata* (Takakura, 1898)^c^	–	AJ436877	–	–	AJ436822	AJ436932	Thollesson and Norenburg (2003)
* Kulikovia manchenkoi * Chernyshev et al., 2018 ^d^	JF293035	HQ856856	JF277730	JF277683	JF277572	HQ848574	Andrade et al. (2012)
*Lineusacutifrons* Southern, 1913	JF304778	HQ856855	JF277727	JF277681	JF277573	GU590937	Andrade et al. (2012)
*Lineusbilineatus* (Renier, 1804)	JF293041	HQ856844	JF277731	JF277682	JF277571	–	Andrade et al. (2012)
*Lineuslacteus* (Rathke, 1843)^e^	JF293065	HQ856850	JF277725	JF277656	JF277584	HQ848583	Andrade et al. (2012)
*Lineuslongissimus* (Gunnerus, 1770)	–	AJ436880	–	–	AJ436825	AJ436935	Thollesson and Norenburg (2003)
*Lineusruber* (Müller, 1774)^f^	JF293040	HQ856853	JF277718	JF277655	JF277583	HQ848580	Andrade et al. (2012)
*Lineussanguineus* (Rathke, 1799)^g^	KF935301	KF935357	KF935413	*	KF935468	KF935518	Kvist et al. (2014)
*Maculauraalaskensis* (Coe, 1901a)^h^	–	AJ436882	AJ436981	–	AJ436827	AJ436937	Thollesson and Norenburg (2003)
*Micrurachlorapardalis* Schwartz & Norenburg, 2005	KF935292	KF935348	KF935404	*	KF935459	KF935512	Kvist et al. (2014)
*Micruradellechiajei* (Hubrecht, 1879)	KF935294	KF935350	KF935406	*	KF935461	KF935514	Kvist et al. (2014)
*Micrurafasciolata* Ehrenberg, 1828	JF293038	HQ856846	JF277721	JF277660	JF277585	HQ848577	Andrade et al. (2012)
*Micruraignea* Schwartz & Norenburg, 2005	JF293043	HQ856859	JF277734	JF277664	JF277588	HQ848587	Andrade et al. (2012)
*Micrurapurpurea* (Dalyell, 1853)	JF293036	HQ856845	JF277726	JF277663	JF277577	HQ848586	Andrade et al. (2012)
*Micruraverrilli* Coe, 1901a	KF935288	KF935344	KF935400	*	KF935455	KF935508	Kvist et al. (2014)
*Micrura* sp.^i^	KF935293	KF935349	KF935405	*	KF935460	KF935513	Kvist et al. (2014)
*Notospermusgeniculatus* (Delle Chiaje, 1828)	KF935295	KF935351	KF935407	*	KF935462	–	Kvist et al. (2014)
*Notospermus* sp. 1 (SK76)	KF935296	KF935352	KF935408	*	KF935463	KF935515	Kvist et al. (2014)
*Notospermus* sp. 2 (SK65)	KF935297	KF935353	KF935409	*	KF935464	–	Kvist et al. (2014)
*Notospermus* sp. 3 (SK50)	KF935298	KF935354	KF935410	*	KF935465	KF935516	Kvist et al. (2014)
*Parborlasiacorrugata* (McIntosh, 1876)	JF293037	HQ856851	JF277732	JF277662	JF277578	–	Andrade et al. (2012)
*Parvicirrusdubius* (Verrill, 1879)	–	AJ436885	–	–	AJ436830	AJ436940	Thollesson and Norenburg (2003)
*Pseudomicruraafzelii* Strand & Sundberg, 2011	GU445924	GU445919	–	–	GU445914	GU392013	Strand and Sundberg (2011)
* Riseriullus occultus * Rogers et al., 1993	JF293031	HQ856848	JF277724	JF277679	JF277581	HQ848581	Andrade et al. (2012)
*Tenuilineusbicolor* (Verrill, 1892)	–	AJ436878	AJ436980	–	AJ436823	AJ436933	Thollesson and Norenburg (2003)
*Zygeupoliarubens* (Coe, 1895)	JF293045	HQ856861	JF277735	JF277661	JF277574	HQ848585	Andrade et al. (2012)
*Yininemertespratensis* (Sun & Lu, 1998)	KY274047	KY274069	KY274091	KY274113	KY274025	KY274003	Present study
Outgroup							
*Baseodiscusmexicanus* (Bürger, 1893)	KF935281	KF935337	KF935393	*	KF935449	KF935503	Kvist et al. (2014)
*Baseodiscusunicolor* Stiasny-Wijnhoff, 1925	KF935284	KF935340	KF935396	*	KF935451	KF935505	Kvist et al. (2014)

^a^Left unidentified in Andrade et al. (2012); the species identification herein follows that of Kajihara et al. (2016)^b^Identified as *Cerebratulusleucopsis* (Coe, 1901b) in Kvist et al. (2014); re-examination of the voucher material at the Museum of Comparative Zoology at Harvard University (MCZ IZ 135331) revealed that it had a branched proboscis (Gonzalo Giribet, pers. comm. to HK) ^c^Transferred to *Kulikovia* by Chernyshev et al. (2018)^d^Identified as *Lineustorquatus* Coe, 1901a in Andrade et al. (2012); the identification herein follows that of Chernyshev et al. (2018)^e^Identified as *Ramphogordiuslacteus* in Andrade et al. (2012); its generic affiliation follows that of Ament-Velásquez et al. (2016)^f^Identified as *Ramphogordiussanguineus* in Andrade et al. (2012); the identification herein follows that of Kang et al. (2015)^g^Identified as *Lineus* sp. in Kvist et al. (2014); the species identification herein follows that of Kang et al. (2015), and its generic affiliation follows that of Ament-Velásquez et al. (2016)^h^Transferred to *Maculaura* by Hiebert and Maslakova (2015)^i^Identified as *Micrurarubramaculosa* Schwartz and Norenburg, 2005 in Kvist et al. (2014); the species identification has been corrected at the database of the Museum of Comparative Zoology at Harvard University for the voucher specimen (MCZ IZ 132531).

PartitionFinder ver. 1.1 (Lanfear et al. 2012) was used to determine the best partition scheme for ML and BI. For BI, the most suitable substitution model for each partition was also selected: GTR+I+G for 16S and 28S; GTR+G for COI (1^st^ codon), H3 (1^st^ and 3^rd^ codons), and H4 (1^st^ and 2^nd^ codons); K80+I+G for 18S and H4 (3^rd^ codon); F81+I+G for COI (2^nd^ codon); HKY+I+G for COI (3^rd^ codon); and JC for H3 (2^nd^ codon). ML analysis was performed using RAxML ver. 8.0.0 (Stamatakis 2014) with a GTR+G model of nucleotide substitution for all partitions consisting of 1000 rapid bootstraps. BI was carried out using MrBayes ver. 3.2.3 (Ronquist and Huelsenbeck 2003; Altekar et al. 2004) with two independent Metropolis-coupled analyses (four Markov chains of 10,000,000 generations for each analysis). Trees were sampled every 100 generations. Values of run convergence indicated that sufficient amounts of trees and parameters were sampled (average standard deviation of split frequencies = 0.006616; minimum estimated sample size of tree lengths = 706.26; potential scale reduction factor of tree lengths = 1.001). Run convergence was also assessed with Tracer ver. 1.6 (Rambaut et al. 2014).

### Population genetics

Using 29 sequences (22 from Korea, seven from China) of 658-bp partial COI gene, haplotype network analyses were performed with Network ver. 5.0.0.1 (available at http://www.fluxus-engineering.com) using median-joining method (Bandelt et al. 1999) and TCS ver. 1.2.1 (Clement et al. 2000) using statistical parsimony (Templeton et al. 1992). Calculation of genetic distances was carried out using MEGA ver. 5.2 (Tamura et al. 2011). Calculations for haplotype diversity, nucleotide diversity, Tajima’s *D*, and Fu’s *F*s values were done with ARLEQUIN ver. 3.5.2.2 (Excoffier et al. 2005).

## Results

### Morphology

The external feature of the Han River nemerteans agreed with the original description of *Y.pratensis* in that these worms were variously dark brown, brick red, and tinged with violet sometimes (Fig. 2A, B). Generally, their body color became paler posteriorly. Sun and Lu (1998) have reported that specimens from the Yangtze River Estuary sometimes show light-red, 4–10 transverse rings arranged on the body. Such ring arrangement was also found in specimens from the Han River Estuary (Fig. 2B) as well as topotype specimens (Fig. 2E, G) collected from muddy sediment with or without vegetation (Fig. 2C, D).

**Figure 3.**
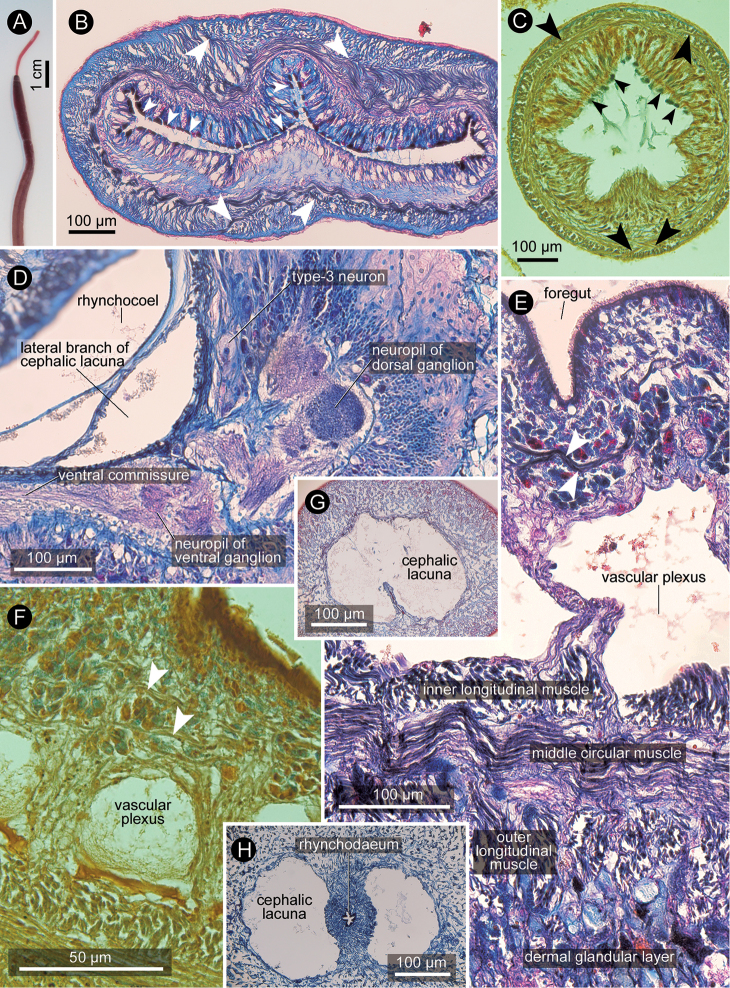
*Yininemertespratensis* (Sun and Lu, 1998), photograph in life (**A**) and photomicrographs of transverse sections (**B, D, E, G, H**ICHUM 5260 **C** DH005C, paratype **F** DH005A, holotype). **A** Anesthetized state with proboscis partially protruded, NIBR IV 0000409596 **B, C** proboscis; large arrow heads indicating fibers contributing to muscle cross; small arrow heads showing rhabdoids **D** cerebral region showing type-3 neuron **E, F** foregut region, arrow heads indicating intra-epithelial somatic muscle fibers **G, H** cephalic region showing well-developed cephalic lacuna.

In specimens collected from the Han River, the proboscis was not branched, and reddish in color (Fig. 3A). Serially sectioned specimen (ICHUM 5260) showed the following internal anatomical features: *i*) the proboscis had two muscle crosses (Fig. 3B), similar to that in the paratype of *Y.pratensis* (Fig. 3C); ii) the rhynchocoel outer circular musculature was not interwoven with the adjacent body-wall longitudinal musculature; iii) the nervous system had type-3 neurons (cf. Beckers 2015) along the inner portion of the brain (Fig. 3D); iv) the foregut wall had intra-epithelial somatic muscle fibres that appeared to be circular or diagonal (Fig. 3E), similar to that observed in the holotype (Fig. 3F); *v*) the body-wall dermal glandular layer was not separated from the body-wall outer longitudinal muscle layer by connective tissue layer (Fig. 3E); and *vi*) the blood system comprised spacious cephalic lacuna (Fig. 3G, H), an alimentary vascular plexus (Fig. 3E), and a mid-dorsal blood vessel.

### Molecular phylogeny

**Figure 4.**
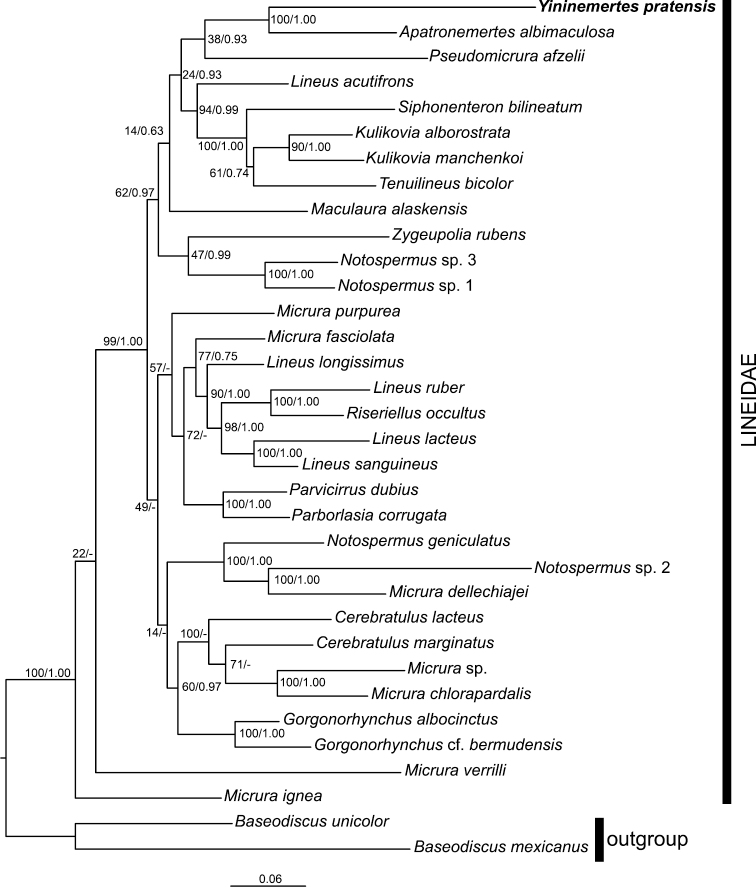
Maximum likelihood tree (ln *L* = −51290.378661) for heteronemerteans based on concatenated 18S rRNA, 28S rRNA, histone H3, histone H4, 16S rRNA, and COI dataset showing phylogenetic position of *Yininemertespratensis* (Sun and Lu, 1998). Numbers near nodes are bootstrap values for maximum-likelihood analysis and posterior probability for Bayesian inference. Scale bar indicates the number of substitutions per site.

Lengths of the six gene markers determined for Korean and Chinese materials were: 16S, 507–508 bp; 18S, 1000–1003 bp; 28S, 1132 bp; COI, 658 bp; H3, 331 bp; and H4, 160 bp. Resulting ML tree (ln *L* = −51290.378661) and BI tree (harmonic mean of estimated marginal likelihood for two runs = −52096.68) were topologically more or less the same, with *Y.pratensis* being a sister of *Apatronemertesalbimaculosa* Wilfert & Gibson, 1974 in both trees with 100% bootstrap support value and 1.0 posterior probability (Fig. 4). The inter-specific K2P distance between the COI sequences of *Y.pratensis* and *A.albimaculosa* was 0.163–0.196. More basal relations between this clade (= *Y.pratensis* + *A.albimaculosa*) and other heteronemerteans included in this analysis were poorly resolved.

### Population genetics

**Figure 5.**
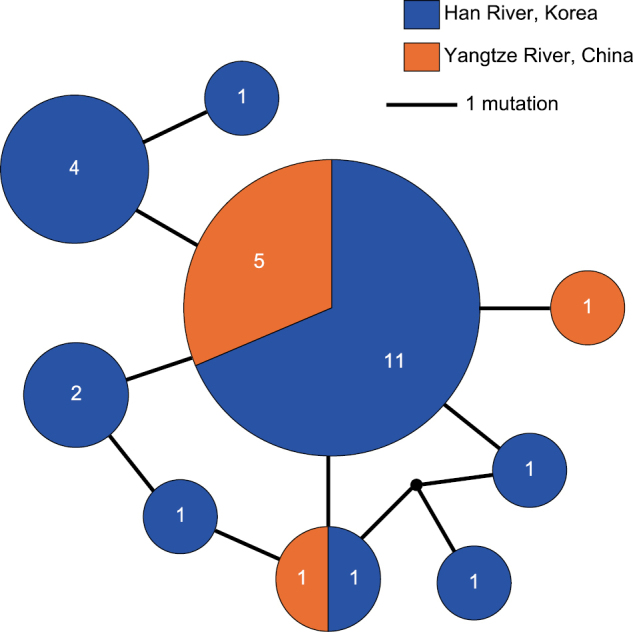
Median-joining network for eight haplotypes detected among 29 *Yininemertespratensis* specimens (22 from Han River, Korea; 7 from Yangtze River, China; statistical-parsimony method yielded the same topology). Numbers in each circle (pie chart) indicate sample size which is proportional to the size of each pie diagram.

Median-joining and statistical parsimony networks were identical in shape, comprising eight haplotypes with a maximal difference of five mutations (Fig. 5). From 29 specimens analysed (22 from Korea, seven from China), a total of nine haplotypes were detected, of which two were shared by Korean and Chinese populations. Eleven of 22 sequences from Korea were represented by the same haplotype, which was also the main haplotype among the Chinese population (shared by five of seven Chinese individuals analysed). Eight COI haplotypes from Korea differed by 0.000–0.006 from each other in terms of both uncorrected *p*-distance and K2P. The Korean population showed higher values of nucleotide diversity and haplotype diversity than the Chinese ones (Table 4). Tajima’s *D* and Fu’s *F*s values were all negative for the Korean population, the Chinese population, and the total population, although not significantly different from zero except for the Fu’s *F*s values for the Korean population and total population.

**Table 4.** Number of individuals analysed for population genetic analysis, number of haplotypes, nucleotide diversity, haplotype diversity, Tajima’s *D*, and Fu’s *F*s based on 658-bp partial COI gene sequences from populations of *Yininemertespratensis* (Sun and Lu, 1998) in the Han River and Yangtze River Estuaries.

Locality	Number of individuals	Number of haplotypes	Nucleotide diversity (S.D.)	Haplotype diversity (S.D.)	Tajima’s *D*	Fu’s *F*s
Han River Estuary, Korea	22	8	0.001849 (0.001365)	0.7316 (0.0897)	−0.80	−3.87*
Yangtze River Estuary, China	7	3	0.000868 (0.000907)	0.5238 (0.2086)	−1.23	−0.92
total	29	9	0.001632 (0.001234)	0.6847 (0.0899)	−1.18	−5.08**

*P = 0.003; **P = 0.000

## Discussion

Because fundamental biological aspects of *Y.pratensis* such as diet, life duration, breeding season, reproductive strategy (semelparous/iteroparous) and mode (oviparous, viviparous, and ovoviviparous), and larval ecology (if the species produces larvae in the first place) are unknown, the causes for the *Y.pratensis* outbreaks since 2013 in the Han River Estuary, South Korea, are open to speculation. One of the potential factors conceivable to explain the *Yininemertes* outbreaks is that the species might be capable of asexual reproduction. Until recently, asexual reproductive capacity among Heteronemertea had been confirmed only in the lineid *Lineussanguineus* (Rathke, 1799) and *L.pseudolacteus* (Gontcharoff, 1951) (cf. Ament-Velásquez et al. 2016). However, asexual reproduction by fragmentation in the valenciniid *Baseodiscusdelineatus* (Delle Chiaje, 1825) (Ikenaga et al. 2019) and *B.hemprichii* (Ehrenberg, 1831) (Kajihara and Hookabe 2019), as well as head-regenerative ability in the lineid *Cerebratuluslineolatus* Coe, 1905 (Zattara et al. 2019), *Lineuspictifrons* Coe, 1904 (Coe 1932), and *L.rubescens* Coe, 1904 (Coe 1930), have been documented. Asexual reproductive capacity may have evolved in more lineages than previously thought among heteronemerteans, possibly including *Y.pratensis*. Another hypothesis is that the Han River ribbon worms might have been introduced from other, unidentified localities. However, this hypothesis sounds rather unlikely, because the haplotype diversity in the Korean population (0.7316), which was greater than the Chinese one (0.5238), suggests that a stable population have existed in the Han River Estuary, probably since long before the first bloom observed in 2013. While Tajima’s *D* and Fu’s *F*s values were overall negative, we cannot draw any robust conclusion about the population dynamics because most of the values were statistically not significant. Future study is needed to pinpoint possible environmental factors that are responsible for the *Y.pratensis* outbreaks, as well as to elucidate the species’ basic biology for obtaining countermeasures against the economic loss to local glass-eel fisheries caused by such blooms.

The family Lineidae McIntosh, 1874 currently contains about 90 genera and 370 species of heteronemerteans, which are morphologically characterized by having horizontal lateral cephalic slits and three apical organs. Most are marine, but six species (each in a monotypic genus) have been described from freshwater or brackish-water habitat. These are *Planolineusexsul* Beauchamp, 1928 from Indonesia; *Siolineusturbidus* Du Bois-Reymond Marcus, 1948 from Amazon; *Hinumanemerteskikuchii* Iwata, 1970 from Japan; *A.albimaculosa* from freshwater tanks in Germany (Wilfert and Gibson 1974), Austria (Senz 1993), USA (Smith 2001), and Japan (Kajihara et al. 2016), as well as in submerged logs and rocks in a pond in Panama (Kvist et al. 2018); *Amniclineuszhujiangensis* Gibson & Qi, 1991 from Zhujiang, China; and *Y.pratensis* from China and Korea (Sun and Lu 1998; present study). Our phylogenetic tree indicates that *A.albimaculosa* and *Y.pratensis* form a highly supported clade, suggesting that the remaining fresh- and brackish-water forms, especially those in Southeast and East Asia, may also belong to the same clade. At this moment, however, neither morphological nor molecular synapomorphy between *A.albimaculosa* and *Y.pratensis* can be perceived; for instance, the characteristic outer cephalic vessels in *A.albimaculosa* are not found in *Y.pratensis*. Both species are reddish in body color, but this may be due to convergent evolution, as freshwater monostiliferous hoplonemerteans in the genus *Prostoma* Dugès, 1828 also possess reddish body. Future studies with expanded taxon sampling, along with detailed morphological examination, should clarify the evolution of these freshwater heteronemerteans.

## References

[B1] Altekar G Dwarkadas S Huelsenbeck JP Ronquist F (2004) Parallel metropolis-coupled Markov chain Monte Carlo for Bayesian phylogenetic inference. Bioinformatics 20: 407–415. 10.1093/bioinformatics/btg427 14960467

[B2] Ament-Velásquez SL Figuet E Ballenghien M Zattara EE Norenburg JL Fernández-Álvarez FA Bierne J Bierne N Galtier N (2016) Population genomics of sexual and asexual lineages in fissiparous ribbon worms (*Lineus*, Nemertea): hybridization, polyploidy and the Meselson effect. Molecular Ecology 25: 3356–3369. 10.1111/mec.13717 27286413

[B3] Andrade SCS Strand M Schwartz M Chen H-X Kajihara H von Döhren J Sun S-C Junoy J Thiel M Norenburg JL Turbeville JM Giribet G Sundberg P (2012) Disentangling ribbon worm relationships: multi-locus analysis supports traditional classification of the phylum Nemertea. Cladistics 28: 141–159. 10.1111/j.1096-0031.2011.00376.x 34872187

[B4] Asakawa M Ito K Kajihara H (2013) Highly toxic ribbon worm *Cephalothrixsimula* containing tetrodotoxin in Hiroshima Bay, Hiroshima Prefecture, Japan. Toxins 5: 376–395. 10.3390/toxins5020376 23430577 PMC3640541

[B5] Bandelt HJ Forster P Röhl A (1999) Median-joining networks for inferring intraspecific phylogenies. Molecular Biology and Evolution 16: 37–48. 10.1093/oxfordjournals.molbev.a026036 10331250

[B6] Beauchamp P de (1928) Une hétéronémerte d’eau douce à Buitenzorg. Bulletin de la Société Zoologique de France 53: 62–67.

[B7] Beckers P (2015) The nervous systems of Pilidiophora (Nemertea). Zoomorphology 134: 1–24. 10.1007/s00435-014-0246-3

[B8] Bourque D Miron G Landry T (2001) Predation on soft-shell clams (*Myaarenaria*) by the nemertean *Cerebratuluslacteus* in Atlantic Canada: implications for control measures. Hydrobiologia 456: 33–44. 10.1023/A:1013061900032

[B9] Bourque D Miron G Landry T (2002) Predatory relationship between the nemertean *Cerebratuluslacteus* and the soft-shell clam, *Myaarenaria*: surface-exploration activity and qualitative observations on feeding behaviour. Canadian Journal of Zoology 80: 1204–1211. 10.1139/z02-095

[B10] Bürger O (1893) Südgeorgische und andere exotische Nemertinen. Zoologische Jahrbücher, Abteilung für Systematik, Ökologie und Geographie der Thiere 7: 207–240. 10.5962/bhl.part.24065

[B11] Chang Y-LK Miyazawa Y Miller MJ Tsukamoto K (2018) Potential impact of ocean circulation on the declining Japanese eel catches. Scientific Reports 8: 5496. 10.1038/s41598-018-23820-6 PMC5883023 29615739

[B12] Chen J-Z Huang S-L Han Y-S (2014) Impact of long-term habitat loss on the Japanese eel *Anguillajaponica*. Estuarine, Coastal and Shelf Science 151: 361–369. 10.1016/j.ecss.2014.06.004

[B13] Chernyshev AV Polyakova NE Turanov SV Kajihara H (2018) Taxonomy and phylogeny of *Lineustorquatus* and allies (Nemertea, Lineidae) with descriptions of a new genus and a new cryptic species. Systematics and Biodiversity 16: 55–68. 10.1080/14772000.2017.1317672

[B14] Clement M Posada D Crandall KA (2000) TCS: a computer program to estimate gene genealogies. Molecular Ecology 9: 1657–1659. 10.1046/j.1365-294x.2000.01020.x 11050560

[B15] Coe WR (1895) Descriptions of three new species of New England palaeonemerteans. Transactions of the Connecticut Academy of Arts and Sciences 9: 515–522. https://www.biodiversitylibrary.org/item/112405#page/559/mode/1up

[B16] Coe WR (1901a) Papers from the Harriman Alaska Expedition. XX. The nemerteans. Proceedings of the Washington Academy of Sciences 3: 1–110. https://www.biodiversitylibrary.org/item/35746#page/21/mode/1up

[B17] Coe WR (1901b) The nemerteans of Porto Rico. Bulletin of the United States Commission of Fish and Fisheries for 1900 2: 223–229.

[B18] Coe WR (1904) Nemerteans of the Pacific coast of North America. Part II. Harriman Alaska Series 11: 111–220. https://www.biodiversitylibrary.org/item/71820#page/159/mode/1up

[B19] Coe WR (1905) Nemerteans of the west and northwest coasts of America. Bulletin of the Museum of Comparative Zoology at Harvard College 47: 1–318. https://www.biodiversitylibrary.org/item/21167#page/5/mode/1up

[B20] Coe WR (1930) Asexual reproduction in nemerteans. Physiological Zoology 3: 297–308. 10.1086/physzool.3.3.30151102

[B21] Coe WR (1932) Regeneration in nemerteans. III. Regeneration in *Lineuspictifrons*. Journal of Experimental Zoology 61: 29–43. 10.1002/jez.1400610104

[B22] Dalyell JG (1853) The Powers of the Creator Displayed in the Creation; or, Observations on Life Amidst the Various Forms of the Humbler Tribes of Animated Nature, Volume II. Van Voorst, London, xiii + 359 pp. https://www.biodiversitylibrary.org/item/62265#page/7/mode/1up

[B23] Delle Chiaje S (1825) Memorie sulla storia e notomia degli animali senza vertebre del regno di Napoli, Volume II. Societa’ Tipografica, Napoli, 185–444. https://www.biodiversitylibrary.org/item/40280#page/7/mode/1up

[B24] Delle Chiaje S (1828) Memorie sulla storia e notomia degli animali senza vertebre del regno di Napoli, Volume III. Societa’ Tipografica, Napoli, xx + 232 pp. https://www.biodiversitylibrary.org/item/40491#page/7/mode/1up

[B25] Du Bois-Reymond Marcus E (1948) An Amazonian heteronemertine. Boletim da Faculdade de Filosofia, Ciências e Letras, Universidade de São Paulo 13: 93–109. 10.11606/issn.2526-4877.bsffclzoologia.1948.125310

[B26] Dugès A (1828) Recherches sur l’organisation et les moeurs des Planariées. Annales des Sciences Naturelles 15: 139–183. 10.5962/bhl.part.18554

[B27] Ehrenberg CG (1828–1831) Phytozoa turbellaria Africana et Asiatica in Phytozoorum Tabula IV et V delineata. In: Hemprich FG Ehrenberg CG (Eds) Symbolae physicae, seu icones et descriptiones corporum naturalium novorum aut minus cognitorum quae ex itineribus per Libyam, Aegyptium, Nubiam, Dongalam Syriam, Arabiam et Habessiniam, pars zoologica II, animalia evertebrata exclusis insectis.Officina Academica, Berolina, 53–67. [pls IV–V] 10.5962/bhl.title.107403 [plates published in 1828, text in 1831]

[B28] Excoffier L Laval G Schneider S (2005) Arlequin (version 3.0): an integrated software package for population genetics data analysis. Evolutionary Bioinformatics Online 1: 47–50. 10.1177/117693430500100003 PMC2658868 19325852

[B29] Gibson R (1994) Nemerteans. Field Studies Council, Shrewsbury, 224 pp.

[B30] Gibson R (1995) Nemertean genera and species of the world: an annotated checklist of original names and description citations, synonyms, current taxonomic status, habitats and recorded zoogeographic distribution. Journal of Natural History 29: 271–561. 10.1080/00222939500770161

[B31] Gibson R Qi S (1991) A new freshwater heteronemertean from the Zhujian (Pearl River), People’s Republic of China. Hydrobiologia 220: 167–178. 10.1007/BF00006550

[B32] Gontcharoff M (1951) Biologie de la régénération et de la reproduction chez quelques Lineidae de France. Annales des Sciences Naturelles, Zoologie, Série 11 13: 149–235.

[B33] Gunnerus JE (1770) Nogle smaa rare og messtendeelen nye Norske Soedyr. Skrifter som udi det Kjobenhavnske Selskab 10: 166–176.

[B34] Hiebert TC Maslakova SA (2015) Integrative taxonomy of the *Micruraalaskensis* Coe, 1901 species complex (Nemertea: Heteronemertea), with descriptions of a new genus *Maculaura* gen. nov. and four new species from the NE Pacific. Zoological Science 32: 615–637. 10.2108/zs150011 26654045

[B35] Hubrecht AAW (1879) The genera of European nemerteans critically revised, with description of several new species. Notes from the Leyden Museum 1: 193–232. https://www.biodiversitylibrary.org/item/37312#page/205/mode/1up

[B36] Ikenaga J Hookabe N Kohtsuka H Yoshida M Kajihara H (2019) A population without female: males of *Baseodiscusdelineatus* (Nemertea: Heteronemertea) reproduce asexually by fragmentation. Zoological Science. [in press; early view available at https://zdw.zoology.or.jp/EarlyView?p=2] 10.2108/zs180203 34664906

[B37] Iwata F (1970) On the brackish water nemerteans from Japan, provided with special circulatory and nephridial organs useful for osmoregulation. Zoologischer Anzeiger 184: 133–154.

[B38] Kajihara H (2014) An objective junior synonym of a ribbon-worm genus name (Nemertea: Heteronemertea). Munis Entomology & Zoology 9: 588. http://www.munisentzool.org/yayin/vol9/issue1/vol9issue1-3895227.pdf

[B39] Kajihara H (2015) A histology-free description of the branched-proboscis ribbonworm *Gorgonorhynchusalbocinctus* sp. nov. (Nemertea: Heteronemertea). Publications of the Seto Marine Biological Laboratory 43: 92–102. 10.5134/199852

[B40] Kajihara H Chernyshev AV Sun S-C Sundberg P Crandall FB (2008) Checklist of nemertean genera and species published between 1995 and 2007. Species Diversity 13: 245–274. 10.12782/specdiv.13.245

[B41] Kajihara H Hookabe N (2019) Anterior regeneration in *Baseodiscushemprichii* (Nemertea: Heteronemertea). Tropical Natural History 19: 39–42. https://www.tci-thaijo.org/index.php/tnh/article/view/181201/128534

[B42] Kajihara H Kuris AM (2013) *Ovicidesparalithodis* (Nemertea, Carcinonemertidae), a new species of symbiotic egg predator of the red king crab *Paralithodescamtschaticus* (Tilesius, 1815) (Decapoda, Anomura). Zookeys 258: 1–15. 10.3897/zookeys.258.4260 PMC3591753 23653496

[B43] Kajihara H Takibata M Grygier MJ (2016) Occurrence and molecular barcode of the freshwater heteronemertean *Apatronemertesalbimaculosa* (Nemertea: Pilidiophora) from Japan. Species Diversity 21: 105–110. 10.12782/sd.21.2.105

[B44] Kang X-X Fernández-Álvarez FÁ Alfaya JEF Machordom A Strand M Sundberg P Sun S-C (2015) Species diversity of *Ramphogordiussanguineus*/*Lineusruber*-like nemerteans (Nemertea: Heteronemertea) and geographic distribution of *R.sanguineus*. Zoological Science 32: 579–589. 10.2108/zs150064 26654041

[B45] Katoh K Standley DM (2013) MAFFT multiple sequence alignment software version 7: improvements in performance and usability. Molecular Biology and Evolution 30: 772–780. 10.1093/molbev/mst010 23329690 PMC3603318

[B46] Kuris AM Blau SF Paul AJ Shields JD Wickham DE (1991) Infestation by brood symbionts and their impact on egg mortality of the red king crab, *Paralithodescamtschatica*, in Alaska: geographic and temporal variation. Canadian Journal of Fisheries and Aquatic Sciences 48: 559–568. 10.1139/f91-071

[B47] Kvist S Laumer CE Junoy J Giribet G (2014) New insight into the phylogeny, systematics and DNA barcoding of Nemertea. Invertebrate Systematics 28: 287–308. 10.1071/IS13061

[B48] Kvist S Chernyshev AV Giribet G (2015) Phylogeny of Nemertea with special interest in the placement of diversity from Far East Russia and northeast Asia. Hydrobiologia 760: 105–119. 10.1007/s10750-015-2310-5

[B49] Kvist S de Carle D Cornejo A Oceguera-Figueroa A (2018) Biological introductions or native rages: two curious cases of new distributional records in the Panama Canal. BioInvasions Records 7: 237–244. 10.3391/bir.2018.7.3.04

[B50] Kwon Y-S Min S-K Yeon S-J Hwang J-H Hong J-S Shin H-S (2017) Assessment of neuronal cell-based cytotoxicty of neurotoxins from an estuarine nemertean in the Han River Estuary. Journal of Microbiology and Biotechnology 27: 725–730. 10.4014/jmb.1611.11027 28081357

[B51] Lanfear R Calcott B Ho SYW Guindon S (2012) PartitionFinder: combined selection of partitioning schemes and substitution models for phylogenetic analyses. Molecular Biology and Evolution 29: 1695–1701. 10.1093/molbev/mss020 22319168

[B52] Lee J-H (2015) Multitude of ribbon worms shock fishermen. The Korea Times. http://www.koreatimes.co.kr/www/news/nation/2015/04/116_176637.html

[B53] Leidy J (1851) Helminthological contributions – No. 3. Proceedings of the Academy of Natural Sciences of Philadelphia 5: 239–244. https://www.biodiversitylibrary.org/item/17633#page/265/mode/1up

[B54] Linnaeus C (1758) Systema naturae per regna tria naturae, secundum classes, ordines, genera, species, cum characteribus, differentiis, synonymis, locis. Tomus I. Editio Decima, Reformata (Volume 1, 10^th^ Edition). Laurentius Salvius, Holmia, xii + 500 pp. 10.5962/bhl.title.542

[B55] Loher T Armstrong DA (2005) Historical changes in the abundance and distribution of ovigerous red king crabs (*Paralithodescamtschaticus*) in Bristol Bay (Alaska), and potential relationship with bottom temperature. Fisheries Oceanography 14: 292–306. 10.1111/j.1365-2419.2005.00337.x

[B56] McIntosh WC (1873–1874) A Monograph of the British Annelids. Part I. The Nemerteans. Ray Society, London. [pp. 1–96, pls I–X published in 1873; pp. 97–214, pls XI–XXIII in 1874] 10.5962/bhl.title.54725

[B57] McIntosh WC (1876) Descriptions of some new species of Annelida from Kerguelen’s Island. Annals and Magazine of Natural History, Series 4 17: 318–323. 10.1080/00222937608681956

[B58] Müller OF (1774) Vermium terrestrium et fluviatilium, seu animalium infusoriorum, helminthicorum, et testaceorum, non marinorum, succincta historia. Volumen Alterum (Volume 2). Heineck and Faber, Havnia et Lipsia, xxxv + 214 pp. 10.5962/bhl.title.12733

[B59] Noh S-H (2019) ‘Ribbon worms’ appear in the Han River Estuary “this year again”. Yonhap News Agency [in Korean]. https://n.news.naver.com/article/001/0010745673

[B60] Özdikmen H (2009) Substitute names for two preoccupied genera (Orthoptera: Acrididae and Tettigonidae). Munis Entomology & Zoology 4: 606–607. http://www.munisentzool.org/yayin/vol4/issue2/606-607.pdf

[B61] Puerta P Andrade SCS Junoy J (2010) Redescription of *Lineusacutifrons* Southern, 1913 (Nemertea: Pilidiophora) and comments on its phylogenetic position. Journal of Natural History 44: 2363–2378. 10.1080/00222933.2010.504895

[B62] Rambaut A Suchard MA Xie D Drummond AJ (2014) Tracer ver. 1.6. http://beast.bio.ed.ac.uk/Tracer

[B63] Rathke H (1843) Beiträge zur Fauna Norwegens. Verhandlungen der Kaiserlichen Leopoldinisch-Carolinischen Akademie der Naturforscher 20: 1–264. 10.5962/bhl.title.120119

[B64] Rathke J (1799) Jagttagelser henhörende til Indvoldeormenes og Blöddyrenes Naturhistorie. Skrivter af Naturhistorie Selskabet, Kjobenhaven 5: 61–148. https://babel.hathitrust.org/cgi/pt?id=hvd.32044106425135;view=1up;seq=1

[B65] Renier SA (1804) Prospetto della classe dei vermi, nominati e ordinati secondo il sistema di Bosc.

[B66] Rogers AD Junoy J Gibson R Thorpe JP (1993) Enzyme electrophoresis, genetic identity and description of a new genus and species of heteronemertean (Nemertea, Anopla) from northwestern Spain and North Wales. Hydrobiologia 266: 219–238. 10.1007/BF00013370

[B67] Ronquist F Huelsenbeck JP (2003) MrBayes 3: Bayesian phylogenetic inference under mixed models. Bioinformatics 19: 1572–1574. 10.1093/bioinformatics/btg180 12912839

[B68] Schwartz ML (2009) Untying a Gordian knot of worms: systematics and taxonomy of the Pilidiophora (phylum Nemertea) from multiple data sets. Ph.D. dissertation, The George Washington University, Washington, D.C.

[B69] Schwartz ML Norenburg JL (2005) Three new species of *Micrura* (Nemertea: Heteronemertea) and a new type of heteronemertean larva from the Caribbean Sea. Caribbean Journal of Science 41: 528–543.

[B70] Senz W (1993) Nemertinen europäischer Küstenbereiche (Nebst ergänzenden Angaben zur Anatomie von *Apatronemertesalbimaculosa* Wilfert & Gibson, 1974). Annalen des Naturhistorischen Museums in Wien 94/95: 47–145.

[B71] Shields JD Wickham DE Kuris AM (1989) *Carcinonemertesregicides* n. sp. (Nemertea), a symbiotic egg predator from the red king crab, *Paralithodescamtschatica* (Decapoda: Anomura), in Alaska. Canadian Journal of Zoology 67: 923–930. 10.1139/z89-134

[B72] Smith DG (2001) Pennak’s Freshwater Invertebrates of the United States, 4^th^ Edition, Porifera to Crustacea. John Wily & Sons, New York, 664 pp.

[B73] Southern R (1913) Nemertinea. Proceedings of the Royal Irish Academy 31: 1–20.

[B74] Stamatakis A (2014) RAxML version 8: a tool for phylogenetic analysis and post-analysis of large phylogenies. Bioinformatics 30: 1312–1313. 10.1093/bioinformatics/btu033 24451623 PMC3998144

[B75] Stiasny-Wijnhoff G (1925) On a collection of nemerteans from Curaçao. Bijdragen tot de Dierkunde 24: 97–120. 10.1163/26660644-02401007

[B76] Strand M Herrera-Bachiller A Nygren A Kånneby T (2014) A new nemertean species: what are the useful characters for ribbon worm descriptions? Journal of the Marine Biological Association of the United Kingdom 94: 317–330. 10.1017/S002531541300146X

[B77] Strand M Sundberg P (2011) A DNA-based description of a new nemertean (phylum Nemertea) species. Marine Biology Research 7: 63–70. 10.1080/17451001003713563

[B78] Sun S-C Lu J-R (1998) A new genus and species of heteronemertean from the Changjiang (Yangtze) River Estuary. Hydrobiologia 367: 175–187. 10.1023/A:1003284302037

[B79] Sun S-C Lu J-R (2008) *Yininemertes* nom. nov. for preoccupied *Yinia* Sun and Lu, 1998 (Nemertea: Heteronemertea). Species Diversity 13: 187–188. 10.12782/specdiv.13.187

[B80] Sundberg P Saur M (1998) Molecular phylogeny of some European heteronemertean (Nemertea) species and the monophyletic status of *Riseriellus*, *Lineus*, and *Micrura*. Molecular Phylogenetics and Evolution 10: 271–280. 10.1006/mpev.1998.0543 10051380

[B81] Sundberg P Andrade SCS Bartolomaeus T Beckers P von Döhren J Krämer D Gibson R Giribet G Herrera-Bachiller A Junoy J Kajihara H Kvist S Kånneby T Sun S-C Thiel M Turbeville JM Strand M (2016) The future of nemertean taxonomy (phylum Nemertea) – a proposal. Zoologica Scripta 45: 579–582. 10.1111/zsc.12182

[B82] Takakura U (1898) Misaki kinbôsan himomushirui (Nemertine) no bunrui [A classification of the nemerteans of the Misaki region]. Dôbutsugaku Zasshi 10: 38–44, 116–120, 184–187, 331–337, 424–429. [In Japanese]

[B83] Tamura K Peterson D Peterson N Stecher G Nei M Kumar S (2011) MEGA5: molecular evolutionary genetics analysis using maximum likelihood, evolutionary distance, and maximum parsimony methods. Molecular Biology and Evolution 28: 2731–2739. 10.1093/molbev/msr121 21546353 PMC3203626

[B84] Tanu MB Mahmud Y Arakawa O Takatani T Kajihara H Kawatsu K Hamano Y Asakawa M Miyazawa K Noguchi T (2004) Immunoenzymatic visualization of tetrodotoxin (TTX) in *Cephalothrix* species (Nemertea: Anopla: Palaeonemertea: Cephalotrichidae) and *Planocerareticulata* (Platyhelminthes: Turbellaria: Polycladida: Planoceridae). Toxicon 44: 515–520. 10.1016/j.toxicon.2004.06.014 15450926

[B85] Tatsukawa K (2003) Eel resources in East Asia. In: Aida K Tsukamoto K Yamauchi K (Eds) Eel Biology.Springer Japan, Tokyo, 293–300. 10.1007/978-4-431-65907-5_20

[B86] Temminck CJ Schlegel H (1842–1850) Pisces [in 16 volumes]. In: von Siebold PF (Ed.) Fauna Japonica sive Descriptio animalium, quae in itinere per Japoniam, jussu et auspiciis superiorum, qui summum in India Batava imperium tenent, suscepto, annis 1825–1830 collegit, notis, observationibus et adumbrationibus illustravit Ph. Fr. de Siebold. Conjunctis studiis C. J. Temminck et H. Schlegel pro vertebratis atque W de Haan pro invertebratis elaborata. A Arnz, Leiden.

[B87] Templeton AR Crandall KA Sing CF (1992) A cladistic analysis of phenotypic associations with haplotypes inferred from restriction endonuclease mapping and DNA sequence data. III. Cladogram estimation. Genetics 132: 619–633. 1385266 10.1093/genetics/132.2.619 PMC1205162

[B88] Thollesson M Norenburg JL (2003) Ribbon worm relationships: a phylogeny of the phylum Nemertea. Proceedings of the Royal Society B 270: 407–415. 10.1098/rspb.2002.2254 12639321 PMC1691252

[B89] Tilesius WC (1815) De cancris camtschaticis, oniscis, entomostracis et cancellis marinis microscopicis noctilucentibus, cum tabulis IV: aenacis et appendice adnexo de acaris et ricinis camtschaticis. Mémoires de l’Académie Impériale de Science de St. Pétersbourg 5: 331–405.

[B90] Tseng M-C Tzeng W-N Lee S-C (2003) Historical decline in the Japanese eel *Anguillajaponica* in northern Taiwan inferred from temporal genetic variations. Zoological Studies 42: 556–563.

[B91] Tzeng W-N (1997) Short- and long-term fluctuations in catches of elvers of the Japanese eel *Anguillajaponica* in Taiwan. In: Hancock DA Smith DC Grand A Beumer JP (Eds) Developing and Sustaining World Fisheries Resources: the State of Science and Management.2^nd^ World Fisheries Congress Proceedings, CSIRO publishing, Collingwood, Australia, 85–89.

[B92] Verrill AE (1879) Notice of recent additions to the marine Invertebrata, of the northeastern coast of America, with descriptions of new genera and species and critical remarks on others. Part I – Annelida, Gephyraea, Nemertina, Nematoda, Polyzoa, Tunicata, Mollusca, Anthozoa, Echinodermata, Porifera. Proceedings of the United States National Museum 2: 165–205. 10.5479/si.00963801.76.165

[B93] Verrill AE (1892) The marine nemerteans of New England and adjacent waters. Transactions of the Connecticut Academy of Arts and Sciences 8: 382–456. https://www.biodiversitylibrary.org/item/32298#page/406/mode/1up

[B94] Wheeler JFG (1940) Notes on Bermudan nemerteans: *Gorgonorhynchusbermudensis*, sp. n. Annals and Magazine of Natural History, Series 11 6: 433–438. 10.1080/03745481.1940.9723699

[B95] Wilfert M Gibson R (1974) A new genus of hermaphroditic freshwater heteronemertean (Nemertinea). Zeitschrift für Morphologie der Tiere 79: 87–112. 10.1007/BF00298777

[B96] Zattara EE Fernández-Álvarez FA Hiebert TC Bely AE Norenburg JL (2019) A phylum-wide survey reveals multiple independent gains of head regeneration in Nemertea Proceedings of the Royal Society B 286: 10.1098/rspb.2018.2524 PMC6458331 30836873

